# Fennel Tea Has a Region‐Specific Effect on the Motility of the Stomach

**DOI:** 10.1111/nmo.70201

**Published:** 2025-11-10

**Authors:** Anita Annahazi, Birgit Kuch, Lejla Ridzal, Nooshin Mansouri, Ida Hosni, Michael Schemann

**Affiliations:** ^1^ Chair of Human Biology Technical University of Munich Freising Germany; ^2^ Chair of Zoology Technical University of Munich Freising Germany

**Keywords:** Ca2+‐channels, fennel tea, functional dyspepsia, spasmolytic, stomach motility

## Abstract

**Background:**

Fennel *(Foeniculum vulgare)*, a culinary and folk medicinal plant used worldwide in infantile colic, bloating, and indigestion, has scarce scientific evidence.

**Methods:**

To explore its impact on gastric motility, guinea pig circular and longitudinal smooth muscle strips from the antrum, corpus, and fundus were mounted in an organ bath, and freshly brewed fennel tea was applied in concentrations from 0.78 to 100 μL mL^−1^. Changes in muscle tone, as well as amplitude and frequency of spontaneous phasic activity, were measured by force transducers. Treatment was repeated in the presence of the neurotoxin tetrodotoxin (TTX), the nitric oxide synthase inhibitor N (ω)‐nitro‐L‐arginine‐methyl ester (L‐NAME), and the nonselective store‐operated Ca2+ entry blocker SKF‐96365.

**Key results:**

Fennel tea did not change the tone or contractile frequency in antrum strips but increased the amplitude of phasic contractions significantly and reversibly at the two highest concentrations in longitudinal and at all concentrations in circular preparations (*p* < 0.05). In the fundus and corpus, it induced a significant, reversible relaxation in both circular and longitudinal muscle strips at all concentrations (*p* < 0.05). TTX and L‐NAME did not alter the effect of fennel tea (*p* > 0.05). However, the relaxation was inhibited by SKF‐96365 in both fundus and corpus (*p* < 0.05).

**Conclusions and Inferences:**

Fennel tea acts as a spasmolytic in the proximal stomach and as a prokinetic in the antrum, which supports its observed beneficial effects in gastrointestinal conditions like functional dyspepsia. The antispasmodic effect seems to be mediated by store‐operated smooth muscle Ca2+ channels, but the exact target needs to be further explored.

Fennel tea has a region‐specific effect on the stomach motility. It relaxes the fundus and the corpus and acts pro‐motility in the antrum. The store operated Ca2+ entry blocker SKF‐96365 hampers the spasmolytic effect of fennel tea in the fundus and corpus. *The image was created using Biorender.*

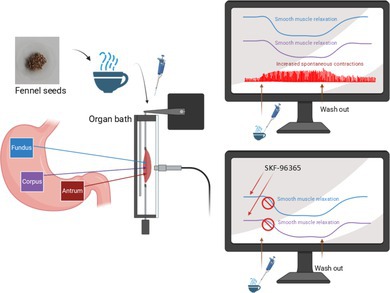

**Summary**

- Fennel tea is a widely used folk remedy for stomach pain, nausea, and bloating with little scientific evidence. We explored its effects on the motility of different parts of the guinea pig stomach in an organ bath.
- Fennel tea caused a relaxation in the fundus and corpus of the stomach, whereas it increased the spontaneous phasic contractions in the antrum. This supports its observed beneficial effects in stomach complaints such as functional dyspepsia.
- The spasmolytic effects in the proximal, but not the prokinetic effects in the distal stomach are likely caused by inhibition of store operated Ca channels expressed by smooth muscle.

## 1 Introduction

Fennel *(Foeniculum vulgare)* is an aromatic plant with culinary and folk medicinal traditions worldwide [1]. In the Middle East, India, Pakistan, and Nepal, fennel seeds are important spices and are also chewed raw after a large meal to freshen the breath and improve digestion [2]. In the traditional medicine of both Eastern and Western cultures, fennel seeds are thought to relieve bloating, nausea, abdominal pain, and infantile colic [3] [4]. Despite its common global use, scientific data on its effects on the gastrointestinal (GI) tract are scarce, and the available studies are mostly nonrandomized, uncontrolled, or apply fennel in combination with other plants.

One part of the literature focuses on the effect of fennel on babies with infantile colic. In one study, fennel tea applied three times, 35 mL daily, significantly reduced the number of crying hours within 1 week [5]. Another study demonstrated a positive effect of a phytotherapeutic agent prepared from *Matricariae recutita L., Foeniculum vulgare M. var. dulce*, and *Melissa officinalis L* extracts, with a reduction of crying hours in over 85% of infantile colic patients treated with the extract [6]. Other studies apply fennel oil made from the seeds. In a double‐blind, randomized, placebo‐controlled trial, fennel oil treatment eliminated infantile colic in 65% of patients, compared to 23.7% in the placebo group [7].

In adult patients, some publications report the use of fennel in irritable bowel syndrome (IBS). In one study, 30 days of treatment with a combination of fennel oil and curcumin extract significantly decreased abdominal pain and distension and improved quality of life, with a good safety profile [8]. Another small pilot study applying the combination of essential fennel oil with turmeric over 2 months showed significant improvement both in symptoms and quality of life in IBS patients [9].

Furthermore, in patients after laparotomic operation for gynecologic malignancies, drinking a cup of fennel tea twice a day enhanced the recovery of intestinal function by significantly reducing the mean time to flatus and defecation, compared to a control group drinking water [10].

Besides the mentioned human studies, which suggest an effect on GI function, there are even fewer data directly exploring changes in motility. In an early study available in full length only in Japanese, fennel oil showed antispasmodic action on mouse small intestines [11]. Furthermore, Digas colic drops (DCD‐684), a polyherbal formulation containing decoctions of five medicinal plants (*Carum carvi L., Foeniculum vulgare Mill, Mentha arvensis L., Mentha piperita L*., and *Zingiber officinale Roscoe*), inhibited spontaneous and KCl, acetylcholine, carbamylcholine, serotonin, and histamine‐induced contractions of isolated rabbit jejunum [12]. However, a German study described increased phasic contractions of guinea pig ileal myenteric plexus‐longitudinal muscle preparations upon fennel oil administration [13].

So far, no studies have explored the effect of fennel on different regions of the stomach systemically. Two publications tested the effect of fennel or its main component, anethole, on the stomach in vivo, with different methodologies. An early study from Japan described the effects of a combined stomachic containing fennel on the spontaneous movement of the unanesthetized rabbit stomach, measured with a balloon implanted in the pyloric antrum muscle [14]. The authors mention that the combined stomachic and some of its components, such as fennel, dose‐dependently accelerated the spontaneous movements. However, the results on fennel are only anecdotal as no data are provided in the manuscript on the effects on motility, nor is it clear which part of fennel was used, that is, the plant itself or its seeds. Another study exploring the effect of anethole, the main component of fennel oil, on gastric emptying in mice showed no effect in the basal state, but a restoration of delayed gastric emptying evoked by clonidine [15]. The latter effect was also confirmed by using fennel oil. Gastric accommodation, measured with a barostat in rats, was potentiated by anethole both in basal conditions and after restraint stress [15].

Tea is a common and commercially easily accessible form of fennel seed treatment, consumed by the general population for various reasons besides gastrointestinal complaints, such as to ameliorate symptoms of a cold [16] or as a galactogen for breastfeeding mothers [17]. Additionally, it has been applied in clinical trials to treat oligo/amenorrhea [18] or to reduce appetite in overweight women [19]. Therefore, we decided to use fennel tea instead of oil or extract in our experiments. As many important components of the tea are volatile, we prepared the tea always freshly from pure seeds during the experiment, provided by the same manufacturer. We were aware of the possible variation in the composition of the freshly made tea and its potential influence on the results. However, we deliberately chose this formulation to mimic the use of fennel in real life as closely as possible.

Based on the scarce results in the literature, our aim was to clarify the effect of fennel tea on the motility of different regions of the stomach.

## 2 Materials and Methods

### 2.1 Animals

Male guinea pigs weighing 280–400 g (Dunkin Hartley, Charles River, Sulzfeld, Deutschland) were kept in isolated airflow units at a temperature of 20°C–24°C and a 14: 10 h light/dark cycle. Standard laboratory food pellets and drinking water were provided *ad libitum*. Animals were euthanized by a blow to the head followed by exsanguination. All animal work was conducted according to the German guidelines for animal care and welfare (Deutsches Tierschutzgesetz) and approved by the Bavarian state ethics committee (Regierung Oberbayern, which serves as the Institutional Care and Use Committee for the Technische Universität München) according to §4 and §11 Deutsches Tierschutzgesetz under reference number 32–568 and 32–568‐2.

### 2.2 Gastric Motility Experiments

After removal, the entire stomach was immersed in ice‐cold, carbogen‐aerated (95% O_2_, 5% CO_2_) Krebs solution (pH 7.4, composition in mmol L^−1^: 117 NaCl, 4.7 KCl, 2.5 CaCl_2_ (2H_2_O), 1.5 MgCl_2_ (6H_2_O), 25 NaHCO_3_, 1.2 NaH_2_PO_4_ and 11.0 Glucose). The stomach was opened from the greater curvature and cut in halves, rinsed in cold Krebs solution, placed in Sylgard‐coated Petri dishes and fixed mucosal side up with metal pins. Under an Olympus SZ51 stereomicroscope (Olympus, Hamburg, Germany), the mucosa was carefully removed. After the removal of the mucosa, four pieces of 1 cm^2^ tissue were cut out from each stomach half: one from the fundus, one from the antrum and (due to its larger size) two from the corpus. Each tissue piece contains the circular and longitudinal muscle layer and the myenteric plexus in between, which allows the assessment of the motility of the smooth muscle layer, under the control of the enteric nervous system, but without the influence of the central nervous system. Each 1 cm^2^ tissue piece was fixed with surgical knots at its two ends, either along the direction of the longitudinal or the circular muscle layer, in order to measure the longitudinal or the circular muscle layer's movements, respectively. Muscle strips were mounted in a four‐chamber, 25 mL automatic organ bath (Panlab, Barcelona, Spain), all chambers were equipped with isometric tension transducers connected with a Quad Bridge and a MacLab/4S analog/digital converter (MacLab, AD Instruments, Spechbach, Germany). During the experiments, the tissue preparations were kept constantly in carbogen‐bubbled Krebs solution at 37°C and pH between 7.3 and 7.4. Changes in force were recorded and analyzed employing LabChart 7 software (MacLab, AD Instruments) on a computer. After setting a preload of 15 mN, an equilibration period was started for 60 min. Electric field stimulation (EFS) was performed with a Grass SD9 stimulator (100 V, 10 Hz, pulse width of 0.5 ms, 10 s) to test tissue viability. Tissues not responding to EFS with a change in tension were discarded.

### 2.3 Drug Treatments

During each experiment, fennel tea was prepared freshly according to the instructions of the manufacturer, namely, 2.5 g of pure fennel seeds were brewed with 150‐mL boiling water (to avoid any potential confounding factors in the tap water, distilled water was used) for 15 min. The seeds were removed by a paper filter, and the tea was allowed to cool down to 38° while being covered. After adding the tea to the organ bath, motility was recorded for at least 10 min, until the tone, or in the case of the antrum, the amplitudes of spontaneous contractions reached a stable value, followed by a washout step. Fennel tea was applied in eight different final concentrations in the organ bath: 0.78, 1.56, 3.13, 6.25, 12.5, 25, 50, and 100 μL mL^−1^. These concentrations had no significant effect on the osmolality or pH of the solution in the chamber. The applications were always applied in a random order, with 3–6 different concentrations per tissue, separated by washouts. Before the next application, enough time was provided for the tone and amplitude to return to baseline. The used concentration range remains below that which would develop after drinking 150 mL of tea on an empty stomach (corresponding to 811 μL mL^−1^ calculated with 35 mL resting water in the fasting stomach [20] or to 130 μL mL^−1^ in case of a stomach already containing 1 L of fluid).

Fennel tea was administered with a combination of different drugs to explore the mechanism of action. If a drug caused a change in the tone, the tone was adjusted to the pre‐drug level before adding fennel tea. Tetrodotoxin (TTX, 1 μM) was administered before fennel tea (100 μL mL^−1^ in the antrum longitudinal and 25 μL mL^−1^ in all other preparations) on preparations from all regions and compared in paired experiments with fennel tea alone. The tested concentration was 100 μL mL‐1 in the antrum longitudinal, because only the 50 and 100 μL mL‐1 concentration of fennel tea had a significant effect, that is, an increase in the spontaneous phasic activity. We used 25 μL mL‐1 in all other preparations, as in all other regions and muscle layers, this concentration was already effective. After checking the reproducibility of fennel tea (using the concentration of 25 μL mL^−1^, because such experiments were performed only in the fundus and corpus, where this concentration was effective, and further experiments with a repeated design also used this concentration) application three times on the same tissue separated by a washout, further pharmacological experiments were performed by using a self‐control design to reduce the number of animals needed. Thus, fennel tea was applied before (followed by a washout) and after the application of the drug of interest, and the first application of fennel tea served as a control. To test the involvement of NO in the relaxation, N (ω)‐nitro‐L‐arginine‐methyl ester (L‐NAME, 100 μM) was added 25 min before fennel tea to fundus and corpus strips and compared to the effect of tea alone. In another set of experiments, to test the role of Ca2+ channels in the relaxation, the non‐selective store‐operated Ca2+ entry blocker SKF‐96365 (10 μM) was added 20 min before fennel tea to the corpus and fundus muscle strips and to antrum circular muscle preparations, and compared to the effect of the tea alone.

### 2.4 Data Analysis

The changes in tone, represented by the alteration in the muscle tension after the application of fennel tea, were compared to the baseline tension before adding tea and expressed as ∆mN. Only the antrum muscle strips presented reliable, regular spontaneous activity; therefore, changes in amplitude and frequency of spontaneous contractions were analyzed only in this region. In the case of paired experiments, a paired Student t‐test was used, or a Wilcoxon signed ranked test in case of data with a non‐Gaussian distribution. For multiple comparisons, one‐way analysis of variance, or in the case of data with a non‐Gaussian distribution, Kruskal–Wallis one‐way analysis of variance on ranks was used. Statistical significance was determined as *p* < 0.05. As not all results were normally distributed, for better comparability, all results, including those with a normal distribution, were presented as median [25%/75%].

## 3 Results

### 3.1 Region‐Specific Effect of Fennel Tea on Motility

**FIGURE 1:**
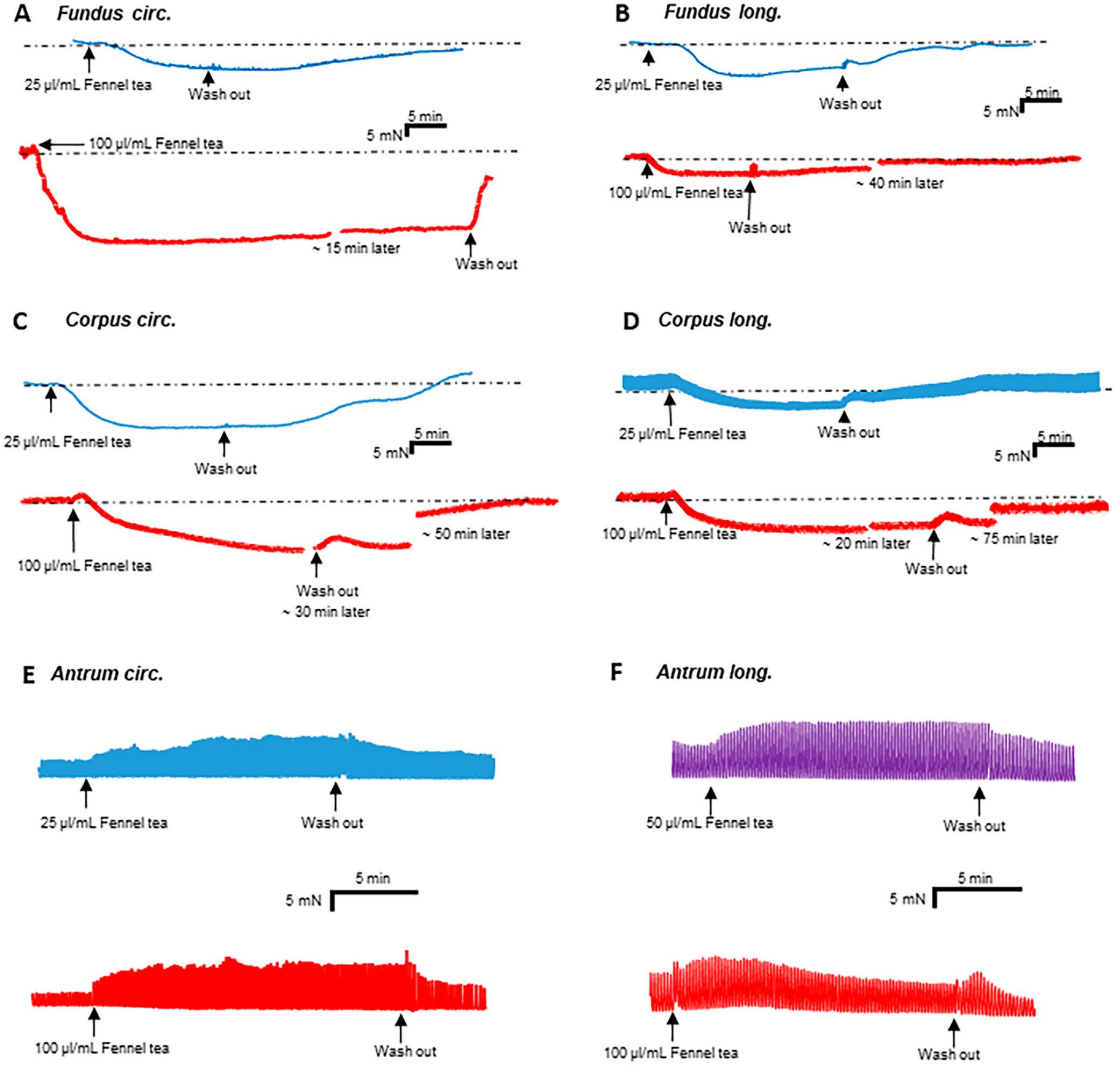
Representative traces of the effect of fennel tea in different regions of the stomach. In the fundus and corpus, fennel tea induces a relaxation, whereas in the antrum, it increases the contraction amplitude of ongoing phasic activity. Depicted are original traces from fundus circular (A) and longitudinal (B), corpus circular (C) and longitudinal (D), and antrum circular (E) and longitudinal (F) muscle strips. In the case of antrum longitudinal, 50 and 100 μL mL^−1^ concentrations are shown, as only these had a significant effect. In the case of all other muscle layers, 25 and 100 μL mL^−1^ concentrations were chosen to demonstrate the effect of fennel tea. In all regions, the effect of fennel tea is reversible by washing out.

**FIGURE 2:**
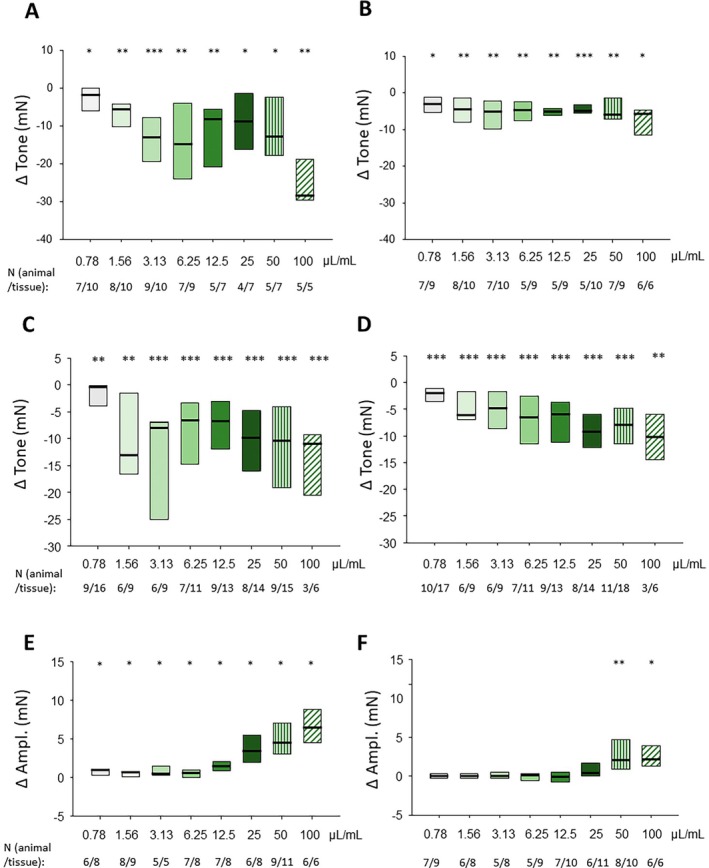
Effects of fennel tea on stomach motility. The differences in gastric smooth muscle tone before and after applying different concentrations of fennel tea are depicted in (A) fundus circular, (B) fundus longitudinal, (C) corpus circular, and (D) corpus longitudinal preparations. (*: *p* < 0.05; **p < 0.01; ***p < 0.001; One‐sample t‐test or signed‐rank test). The difference in the amplitude of spontaneous phasic contractions before and after applying different concentrations of fennel tea is shown in (E) antrum circular and (F) antrum longitudinal preparations. (*: *p* < 0.05; **p < 0.01 One‐sample *t*‐test or signed‐rank test).

In both fundus circular and longitudinal muscle, all concentrations (0.78, 1.56, 3.13, 6.25, 12.5, 25, 50, and 100 μL mL^−1^) induced a significant reduction of muscle tone (Figure 1A,B; Figure 2A,B). This effect was completely reversible by washout.

Likewise, in the corpus circular and longitudinal muscle, all tested concentrations caused a significant relaxation (Figure 1C,D, Figure 2C,D).

In the antrum circular and longitudinal muscle, none of the tested concentrations evoked changes in the muscle tone (*p* > 0.05). However, in the antrum circular muscle preparations, all tested concentrations increased the amplitude of spontaneous contractions significantly (Figure 1E, Figure 2E). In longitudinal muscle strips of the antrum, only the two highest concentrations caused a significant increase (Figures 1F, 2F). The contractile frequency remained unchanged in both circular and longitudinal muscle preparations at all concentrations.

In addition to the raw data, changes in motility were normalized to the response of the EFS. During EFS, all enteric neurons are stimulated, and they release their neurotransmitters to cause an increase as well as a decrease in motility. We calculated the change related to the contractile response to EFS in case of the antrum, and to the relaxation response in case of the fundus and the corpus. The relative values in % align well with the absolute values and are presented in Table 1.

**TABLE 1:** Relative changes in motility in the different stomach regions normalized to the response to electric field stimulation (EFS).

		Concentration of fennel tea (μL mL^−1^) in the organ bath							
		0.78	1.56	3.13	6.25	12.5	25	50	100
% of change related to response to EFS	Fundus circ.	20.4 [−1.8/38.7]*	36.3 [25.8/111.5]*	119.4 [75.8/153.8]***	119.0 [69.4/184.3]**	121.4 [91.6/149.7]***	64.5 [55/125.2]*	114.5 [73.7/223.7]**	189.1 [67.7/248.7]*
	Fundus long.	40.4 [6.0/48.4]**	51.9 [34.2/123.8]**	61.2 [46.4/125.5]**	73.5 [41.8/343.2]**	60.7 [44.0/324.9]**	78.0 [47.1/209.0]**	56.9 [37.9/302.8]**	108.4 [69.8/216.5]*
	Corpus circ.	8.7 [−0.2/51.5]**	96.8 [19.1/248.0]**	121.3 [61.7/290.1]**	117.3 [61.1/265.2]***	158.7 [75.7/238.3]***	155.4 [62.0/222.5]***	204.6 [72.8/281.7]***	179.4 [68.0/371.9]*
	Corpus long.	26.0 [15.4/38.0]***	59.2 [28.0/165.8]**	60.0 [33.5/125.7]**	50.2 [28.2/93.1]**	84.3 [53.1/151.3]***	108.6 [59.3/200.4]***	79.4 [51.5/173.9]***	92.8 [63.0/212.1]*
	Antrum circ.	4.1 [1.0/11.1]**	2.8 [0.3/8.4]*	4.1 [−0.7/7.6]	6.3 [−0.1/12.8]*	13.0 [4.6/20.1]**	28.0 [8.1/46.0]**	30.1 [7.9/40.5]**	11.9 [5.4/23.6]*
	Antrum long.	0.1 [−1.4/3.8]	−0.04 [−4.2/1.7]	0.00 [−1.9/1.6]	1.4 [−1.7/6.5]	−0.2 [−3.8/5.6]	3.3 [−3.8/9.7]	11.5 [6.9/18.1]**	7.5 [2.9/18.2]*

*Note:* In fundus and corpus, relaxation is expressed as relative change in % compared to the maximal relaxation response to EFS in a given muscle strip. In antrum, contractile changes are expressed as relative change in % compared to the maximal contractile response to EFS in a given muscle strip. Results are presented as median [25/75].

* *p* < 0.05.

** *p* < 0.01.

*** *p* < 0.001.

### 3.2 Reversibility

All effects were reversible at all examined doses after performing a wash out. In some tissues where 50 and 100 μL mL^−1^ were applied, the wash out step had to be repeated two to three times to fully reverse the effect of fennel tea, whereas at the lower concentrations, one single wash out step was sufficient.

### 3.3 Repeated Administration of Fennel Tea

Repeated administration of 25 μL mL^−1^ fennel tea on the same tissue, separated by wash outs, evoked a similar change in tone in the fundus circular (*n* = 5/5; −13.1 [−13.8/−8.9] vs. −10.6 [−20.4/−7.1] vs. −10.9 [−19.5/−6.8] mN; *p* = 0.954), fundus longitudinal (*n* = 5/9; −5.8 [−8.4/−2.8] vs. −3.4 [−6.7/−3.2] vs. −5.3 [−5.7/−2.3] mN; *p* = 0.971), corpus circular (*n* = 9/9; −6.0 [−8.5/−3.2] vs. −7.1 [−9.6/−2.2] vs. −5.7 [−10.4/−1.8] mN; *p* = 0.569), and corpus longitudinal (*n* = 6/11; −8.0 [−12.6/−5.5] vs. −7.0 [−8.5/−5.1] vs. −6.6 [−11.1/−4.0] mN; *p* = 0.307) preparations.

### 3.4 Pharmacology

**FIGURE 3:**
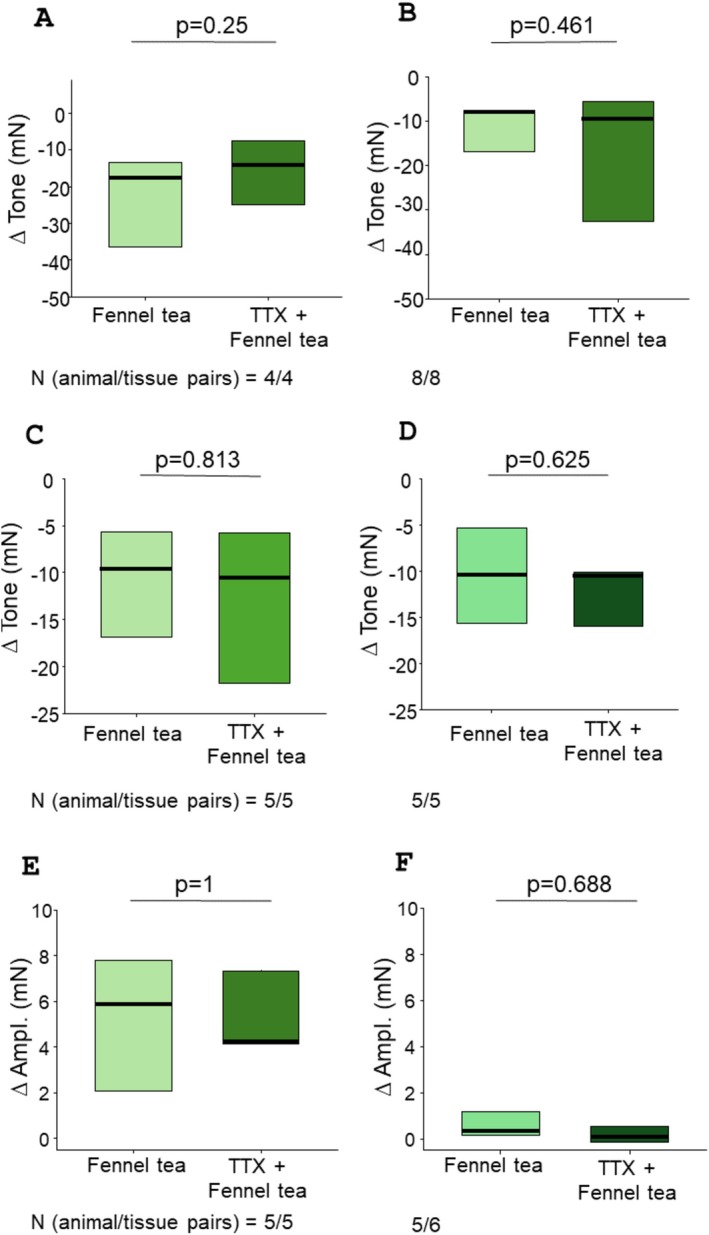
Influence of tetrodotoxin (TTX) on the effect of fennel tea on stomach motility. Difference in gastric smooth muscle tone before and after applying fennel tea (25 μL mL^−1^) with or without previous administration of TTX in (A) fundus circular, (B) fundus longitudinal, (C) corpus circular, and (D) corpus longitudinal preparations (Wilcoxon signed‐rank test). The difference in the amplitude of spontaneous phasic contractions before and after fennel tea (25 μL mL^−1^ for antrum circular and 100 μL mL^−1^ for antrum longitudinal) with or without previous administration of TTX are shown in (E) antrum circular and (F) antrum longitudinal preparations (Wilcoxon signed‐rank test).

TTX did not change the basal tone and had no effect on the relaxation caused by fennel tea in fundus circular (*n* = 4/4; −17.6 [−36.6/−13.3] vs. −14.2 [−25.0/−37.5] mN; *p* = 0.25; Figure 3A), fundus longitudinal (*n* = 8/8; −7.9 [−17.0/−7.4] vs. −9.5 [−32.7/−5.5] mN; *p* = 0.461; Figure 3B), corpus circular (*n* = 5/5; −9.6 [−16.9/−5.7] vs. −10.6 [−21.8/−5.9] mN; *p* = 0.813 Figure 3 C), and corpus longitudinal muscle strips (*n* = 5/5; −10.4 [−15.7/−5.3] vs. −10.6 [−16/−10.1] mN; *p* = 0.625; Figure 3D). Similarly, it did not influence the increase in the amplitude of spontaneous contractions in the antrum circular (*n* = 5/5; 5.8 [2.0/7.8] vs. 4.2 [4.1/7.3] mN, *p* = 1; Figure 3E) and antrum longitudinal (*n* = 5/6; 0.8 [0.2/1.3] vs. 0.6 [0.1/1.4] mN; *p* = 0.688; Figure 3F) preparations.

**FIGURE 4:**
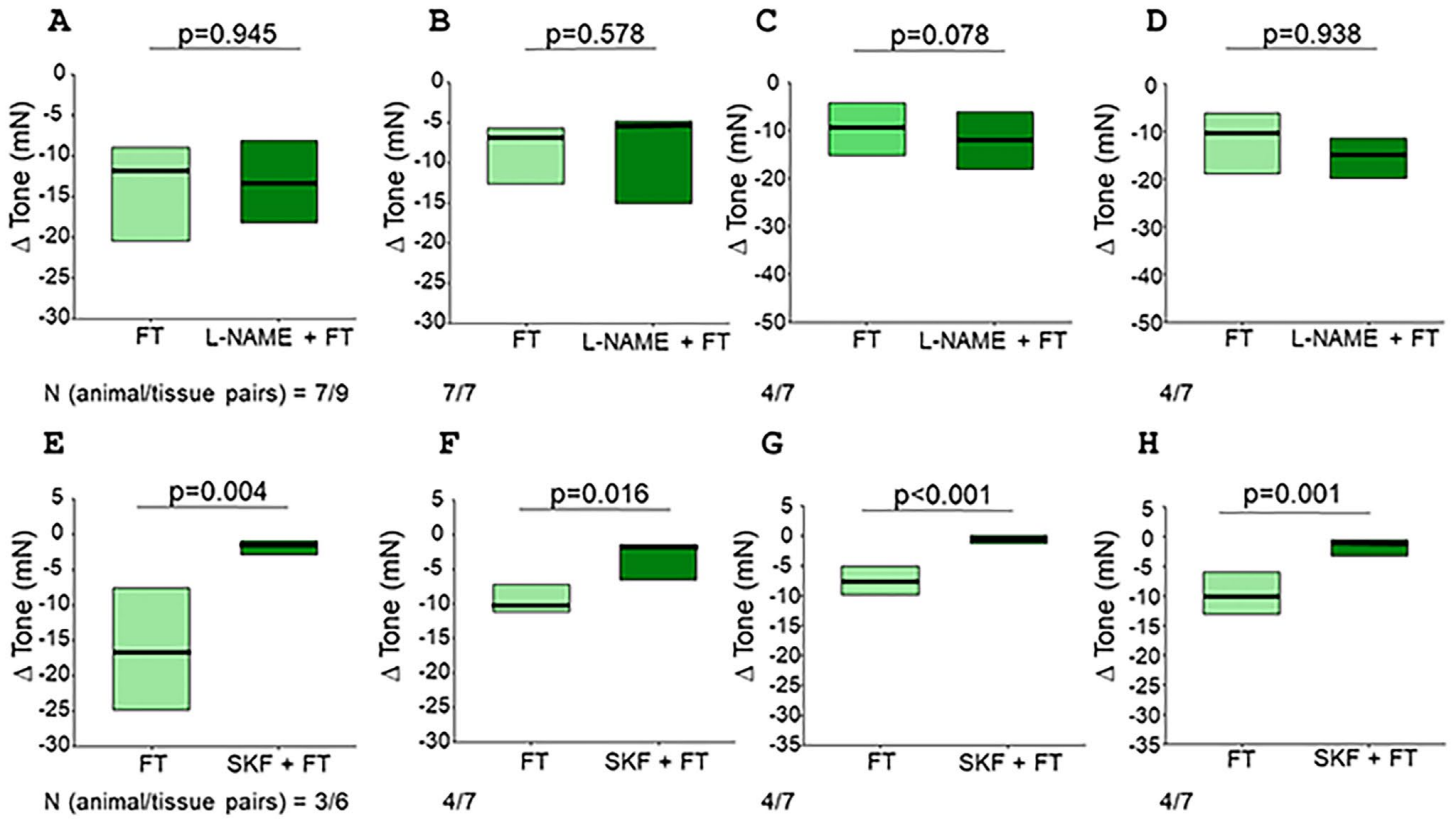
Influence of L‐NAME and SKF‐96365 on the relaxing effect of fennel tea (FT). Difference in gastric smooth muscle tone before and after applying fennel tea (25 μL mL^−1^) with or without previous administration of L‐NAME in (A) fundus circular, (B) fundus longitudinal, (C) corpus circular, and (D) corpus longitudinal preparations (Wilcoxon signed‐rank test). Difference in gastric smooth muscle tone before and after applying fennel tea (25 μL mL^−1^) with or without previous administration of SKF‐96365 (SKF) in (E) fundus circular, (F) fundus longitudinal, (G) corpus circular, and (H) corpus longitudinal preparations (Wilcoxon signed‐rank test).

L‐NAME caused a quick increase in tone within the first 10 min of incubation (peak increase of 13 ± 2.9; 8.1 ± 2.2; 8.8 ± 1.9, and 6.8 ± 1.6 mN in fundus circular, fundus longitudinal, corpus circular and corpus longitudinal preparations, respectively), which started to gradually decrease and was readjusted to the pre‐L‐NAME values at the end of the 20 min incubation period before adding fennel tea if necessary. L‐NAME did not affect the reduction in tone caused by fennel tea in fundus circular (*n* = 7/9; −12.1 [−20.4/−9.0] vs. −13.3 [−18.2/−8.2] mN; *p* = 0.945; Figure 4A), fundus longitudinal (*n* = 7/7; −6.9 [−12.6/−5.6] vs. −5.1 [−15.0/−4.8] mN; *p* = 0.578; Figure 4B), corpus circular (*n* = 4/7; −8.9 [−15.3/−4.4] vs. −12.0 [−18.0/−6.2] mN; *p* = 0.078; Figure 4C), and corpus longitudinal (*n* = 4/7; −10.4 [−18.9/−6.1] vs. −15.1 [−19.8/−11.4] mN; *p* = 0.938; Figure 4D) strips.

**FIGURE 5:**
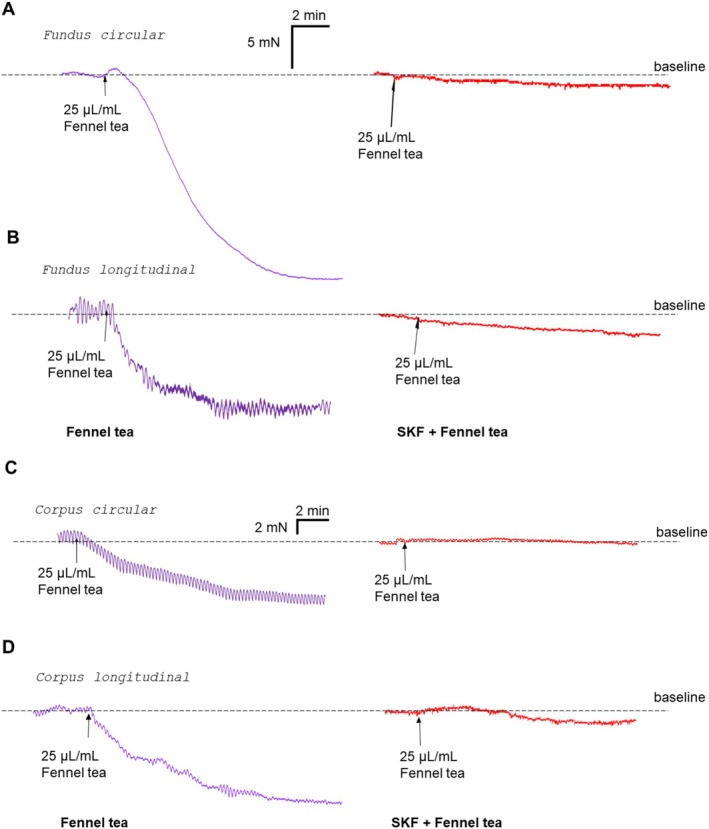
Representative traces of the influence of SKF‐96365 on the relaxing effect of fennel tea in different regions of the stomach. Previous administration of SKF‐96365 (SKF) diminished the relaxing effect of fennel tea, shown on fundus circular (A), fundus longitudinal (B), corpus circular (C), and corpus longitudinal (D) muscle strips.

**FIGURE 6:**
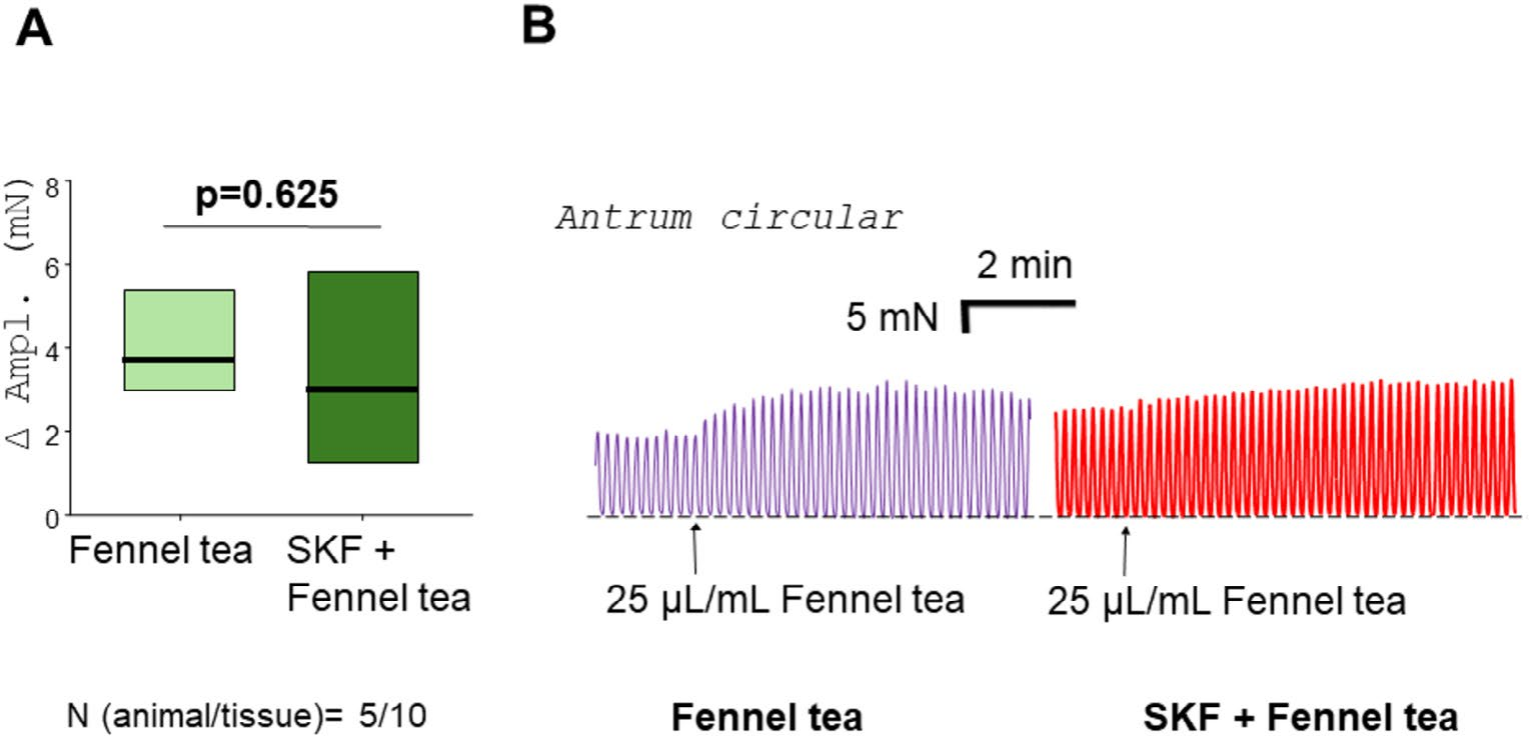
The influence of SKF‐96365 on the effect of fennel tea on the antrum circular muscle. Previous administration of SKF‐96365 (SKF) did not change the increase in the spontaneous activity caused by fennel tea. Paired *t*‐test (A) and representative trace (B).

Upon applying SKF‐96365, the tone decreased rapidly in fundus and corpus (fundus circ.: −22.2 ± 3.4 mN, fundus long.: −6.1 ± 0.6 mN, corpus circ.: −17.6 ± 4.3 mN, corpus long.: −11.0 ± 2.8 mN), and was re‐adjusted to the baseline values before adding fennel tea. SKF‐96365 abolished the relaxation evoked by fennel tea in the fundus circular (*n* = 3/6; −17.1 [−24.9/−7.6] vs. −1.5 [−3.0/−1.0] mN; *p* = 0.004; Figures 4E, 5A), fundus longitudinal (*n* = 4/7; −10.6 [−11.1/−7.2] vs. −2.2 [−6.5/−1.3] mN; *p* = 0.016; Figures 4F, 5B), corpus circular (*n* = 4/7; −7.7 [−9.8/−5.1] vs. −0.7 [−1.3/0.2] mN; *p* < 0.001; Figures 4G, 5C), and corpus longitudinal (*n* = 4/7; −10.3 [−13.2/−5.9] vs. −1.3 [−3.2/−0.6] mN; *p* = 0.001; Figure 4H, Figure 5 D) tissues. In the antrum circular, SKF‐96365 evoked a minimal, transient decrease in tone, peaking at −0.9 ± 0.1 mN, which returned completely to baseline in 4 out of 10 cases. In the remaining six cases, the remaining difference was so small that an adjustment was technically not feasible. Interestingly, SKF‐96365 did not change the increase of amplitude caused by fennel tea in antrum circular muscles (*n* = 5/10; 3.7 [3.0/5.4] vs. 2.9 [1.3/5.8]; *p* = 0.625, Figure 6).

## 4 Discussion

To the best of our knowledge, this is the first report describing the effects of fennel tea on stomach motility in different regions. Fennel tea caused a significant relaxation in the fundus and corpus, which was completely reversible upon washout. In the antrum, fennel tea increased the amplitude of spontaneous phasic contractions, which was also reversible upon washout. However, it had no effect on the basal tone or the frequency of the contractions. Previously, our group reported similar region‐specific effects of papain, a cysteine‐protease of papaya [21], and the herbal preparation STW 5 (Iberogast) [22] using the same recording technique.

In functional dyspepsia patients, one of the main factors in the pathomechanism is disturbed stomach motility. In different studies, impaired gastric accommodation was detected in 15%–50% of patients, which was associated with some of the key symptoms such as early satiation, fullness, and weight loss [23]. In one part of patients, antral hypomotility and delayed gastric emptying to solids and liquids were also described [24]. The observed in vitro effects of fennel tea work in the direction to counterbalance such motility alterations, as it would facilitate the relaxation of the proximal stomach and improve antral motility. In the fundus and antrum circular muscle, the magnitude of the effect of higher concentrations of fennel tea was comparable to that of higher concentrations of STW 5. As the efficacy of STW 5 in alleviating symptoms of functional dyspepsia has been proven by various clinical studies [25], the similarity of the effects supports a possible benefit of fennel tea in this disorder. Using data from a publication analyzing the same tea product as we used in our experiments (fennel tea from Bombastus Werke, Germany) [26], the concentration of trans‐anethole in the human stomach could reach approximately 107 μg mL^−1^ after consumption of a cup (150 mL according to the manufacturer's instructions) of fennel tea on an empty stomach, given that the resting fluid volume is approximately 35 mL [20]. In our experiments, in the case of the 25 μL mL^−1^ end concentration of fennel tea in the organ bath chamber, the concentration of anethole would be around 3.3 μg mL^−1^μg/mL, while in the case of the maximal concentration tested (100 μL mL^−1^ fennel tea), around 12 μg mL^−1^. Trans‐anethole is highly absorbed from the GI tract, considering that > 95% of 14C was recovered from the 0–24 h urine after administration of C^14^ radio‐labeled trans‐anethole in mice and rats, and 54%–69% of the administered dose was excreted in urine and 13%–17% in the breath in healthy human volunteers [27] [28]. This suggests that our tested concentrations can likely be reached in the tissue, making them physiologically relevant.

It seems rational that the observed region‐specific effects are related to different components of fennel, some having antispasmodic and others opposite effects. In case of STW 5, as it is a mixture of nine medicinal plants, it was initially suspected that the different components are responsible for the opposite effects, some causing a contraction, whereas others a relaxation [22]. However, the exploration of the effects of its individual plant extracts revealed that some of them genuinely bear with the same region‐specific effects as STW 5, namely, inhibition in the proximal stomach and contraction‐enhancing properties in the distal stomach [29]. Our studies performed with papain demonstrate that one single chemical compound can influence motility differently, depending on the stomach region [21]. These results suggest that different ion channels or signal transduction pathways are functionally expressed, and activated in the proximal and distal stomach through the examined substance [29]. Region‐specific effects have been also described in the stomach of cows with cinnamaldehyde, an essential oil component of cinnamon, where it relaxed the antrum, but did not change the basal tone of abomasal fundus, and significantly increased the barium chloride‐induced contractions of the latter [30]. The spasmolytic effect of cinnamaldehyde was explained by blockade of Ca2+ channels. One can speculate that herbal components, such as those found in fennel tea or in STW 5, act as a blocker on some Ca2+ channels, which are present in the proximal stomach, causing its relaxation, but are absent in the distal region. Unfortunately, data on differences in calcium‐handling properties of proximal and distal stomach are scarce. It has been shown in the rabbit, that dihydropyridine‐sensitive Ca2+ channels increase in number with postnatal age in the antrum, but not in the fundus [31]. Parallel to this, receptor‐operated bethanechol‐stimulated contraction was completely blocked by the L‐type Ca2+ channel blocker nifedipine in the antrum, but was reduced only by 25% in the fundus, suggesting that smooth muscle in the fundus also uses intracellular Ca2+ sources for this contraction, contrary to the antrum [31]. Moreover, isolated smooth muscle cells from the fundus of cats contracted in response to acetylcholine and 1,4,5‐inositol triphosphate by using intracellular Ca2+ stores, whereas the acetylcholine‐induced contraction also needed extracellular Ca2+ sources in the antrum [32]. These studies suggest that different Ca2+ channels may be involved in smooth muscle contraction of proximal and distal stomach regions, but the exact cause of the opposing effect of fennel tea needs to be explored.

Our experiments with the fast voltage‐gated sodium channel blocker TTX showed no influence on the effect of fennel tea, suggesting that it is likely not nerve‐mediated, although we cannot exclude the role of neural TTX‐insensitive mechanisms. We hypothesized that the spasmolytic effect in the fundus and corpus is either mediated by nitric oxide production or by direct blockage of Ca2+ channels on the smooth muscle. Pretreatment of the tissue with L‐NAME demonstrated that nitric oxide release is not involved in the relaxation. The action of fennel tea on Ca2+ channels was confirmed by SKF‐96365 in the fundus and corpus. SKF‐96365 is a non‐specific Ca2+ channel blocker, which is used in many in vitro and in vivo studies as a blocker of TRP channels and store‐operated Ca2+ entry (SOCE). We have recently demonstrated that the spasmolytic effects of STW 5 are also blocked by SKF‐96365 [33]. In the case of STW 5, our complex pharmacological study has identified ORAI channels as the most likely target, modulated by IP3‐ and PKC‐dependent mechanisms. However, there are some differences between the effects of the two herbal preparations. The magnitude of the relaxation using the higher doses of fennel tea is only comparable to STW 5 in the circular, but not in the longitudinal fundus, where the observed relaxation is much more subtle. Furthermore, fennel tea only affected the longitudinal muscle layer in the antrum at the two highest concentrations, contrary to the circular muscle layer, where it was already effective at much lower concentrations. Therefore, we could not pool muscle strips with different orientations for the statistical analysis, as it was possible in the case of STW 5 or papain, where the effect of the tested substances was identical. Several differences have been described between the circular and longitudinal muscles of the gut [34]. For example, while many receptors are present on the smooth muscle cells of both layers, somatostatin, opioid, and neuropeptide Y (NPY) Y2 and Y4 receptors are absent in the longitudinal muscle layer. Additionally, Ca2+ mobilization requires Ca2+ influx in the longitudinal, but not in the circular muscle in the intestines [35]. Furthermore, in the longitudinal, but not in the circular muscle of the intestines, ryanodine‐sensitive IP3‐insensitive Ca2+ release channels are present, activated by agonist‐mediated Ca2+ influx, resulting in a Ca2+ induced Ca2+ release [36]. The different Ca2+ handling properties of the two layers may explain the observed difference in the effect of fennel tea.

The tone of gastric smooth muscle can also be regulated by Ca2+ sensitization pathways: RhoA/ROCK, which phosphorylates myosin‐phosphatase targeting subunit 1 (MYPT1), and PKC, which phosphorylates protein kinase C (PKC)‐potentiated phosphatase inhibitor protein of 17 kDa (CPI‐17), leading to myofilament Ca2+ sensitization [37]. In the mouse fundus, cholinergic neurotransmission leads to phosphorylation of CPI‐17, whereas carbachol added to the bath activates both CPI‐17 and MYPT1 [38]. Whether fennel also influences these pathways is unknown. However, our data favour Ca2+ entry–dominant mechanism as the effects of fennel tea are almost completely abolished with SKF‐96365, which is used in many studies to distinguish Ca2+ entry–dominant mechanisms from those involving Ca2+ sensitization. Likely, the mechanism of action of fennel tea shows some similarities to STW 5, acting on TRP or SOCE (based on the action of SKF‐96365), but it also has distinct pathways. As SKF‐96365 in some models nonselectively blocks receptor‐mediated and voltage‐gated Ca2+ channels, and K+ channels [39] [40], further studies are needed to clarify which of the potential Ca2+ channel targets of SKF‐96365 abolishes the effect of fennel tea in the proximal stomach. Additionally, a more complex pharmacological exploration should follow to decipher its further targets.

## 5 Conclusions

In summary, we have demonstrated a reversible, region‐specific effect of fennel tea on the stomach in the basal state. While fennel tea caused a relaxation in the proximal stomach, it increased motility in the distal stomach. These effects may support the traditional belief that it improves gastrointestinal complaints such as fullness and nausea. The spasmolytic effects seem to be a calcium‐mediated direct action on the smooth muscle, but the exact Ca2+ channels involved need to be further clarified.

## Author Contributions

A.A.: conceptualization, formal analysis, funding acquisition, investigation, supervision, visualisation, writing – original draft, writing – review and editing. B.K.: investigation, formal analysis. L.R.: investigation, formal analysis, writing – original draft, writing – review and editing. N.M.: investigation, formal analysis. I.H.: investigation, formal analysis. M.S.: conceptualization, resources, supervision, writing – original draft, writing – review and editing.

## Conflicts of Interest

The authors declare no conflicts of interest.

## Data Availability

The data that support the findings of this study are available from the corresponding author upon reasonable request.
